# Arsenicum Album

**Published:** 1887-09

**Authors:** A. McNeil

**Affiliations:** San Francisco, Cal.


### ARSENICUM ALB.—WHITE ARSENIC, ARSENIOUS ACID.

A LECTURE BY PROFESSOR A. MCNEIL, M. D., SAN FRANCISCO, CAL.

This drug has the widest range of application. It is one of the first antipsorics, and is antisycotic and antisyphilitic, thus corresponding to all of Hahnemann's chronic miasms as well as being a leader in the treatment of acute diseases. In them it is adapted to malarial and typhoid fevers, eruptive and inflammatory and pyæmic ones. It also meets the requirements of the diseased symptoms from the new-born babe to the centenarian. Among its attributes are violence and malignity: the pains it cures are violent, and the fevers it relieves are malignant. But do not think our remedy is a panacea. For its indications which limit its benefits are clear cut and sharply defined.

In mental disorders we need it in the delirium of fever and the mania of the lunatic. But one grand characteristic it has in all of these—AN ANGUISH CAUSING RESTLESSNESS. If your patient is apathetic, or not unhappy, you may drop Arsenicum from consideration.

Please remember one point when you find the characteristic of this or any other drug, do not limit its administration to the diseases I name. The totality of the symptoms, not the name of the disease, defines the usefulness of a remedy.

In the delirium of mania-a-potu he hears voices and sees animals, or he is haunted by visions of rats or mice somewhat similar to that of Calc-carb. In suicidal melancholy, with much emaciation and broken health, with Aurum the patient's physical health is good, and this point of difference in the desire to commit suicide of these drugs is that when Arsen. is indicated he is furious, with Aurum he is cunning. As a concomitant of fever, pain, and the like, he is tormented with anguish (Aconite, anxiety), which makes him restless, and he tosses about. (With Rhus he moves because his pains are relieved thereby; with Arnica, because the bed seems hard to the parts on which he lies, but with Arsenic it is because of this anguish of mind; he is not relieved at all, and, in fact, is much exhausted by his efforts.)

He is afraid of death. (Aconite also has this fear, but with it he usually names the day on which he will die.) This anguish and restlessness deprive him of sleep, particularly at three A. M. For the same reason he is afraid of being alone, thus reminding us of Stramon. It is this frame of mind that makes him move about from one bed to another and from one room to another. The patient is tormented by the hallucination that there is one by his side that does all that he is doing, eating, washing, etc.; with Gelsem. he may think that another, not himself, is sick. The headaches cured by Arsenic are, like all the pains of this drug, accompanied by anguish and restlessness, and are relieved by heat and by wrapping up the head warm. (Silic. and Mag. mur. also.)

On the external head we see circular patches denuded of hair; the bare spots are rough and dirty looking. His head is hot, hair sensitive, so that he cannot bear to have it touched.

Of eruptions on the scalp, Arsenic cures dry (seldom moist, and then they are bad smelling and purulent), white, bran-like; burning, itching in the fore part of the head, bleeds when scratched and burns more violently.

In diseases of the eyes this remedy is invaluable. The ophthalmia which indicates Arsen. has violent burning, the lachrymation profuse, corroding the cheeks and all parts with which it comes in contact, and is relieved by warmth. Also ophthalmia with ulcers on cornea before and during the catamenia. Objects appear green (Phosph.), appear as through a white gauze.

Patient has horizontal half sight, only objects in the lower half of the field of vision could be seen (with Aurum he may see either the lower or the upper half). Ophthalmia of children when the skin is rough, dry, and dirty looking, intense pain from the least ray of light, with profuse lachrymation.

The paroxysms of pain are accompanied by roaring in the ears. Patient has hardness of hearing so that he cannot hear the human voice, similar to that of Phosphorus.

Arsenic is indicated in cold in the head and hay fever when there is a watery discharge which burns and excoriates the mucous membrane and skin with which it comes in contact. (Merc.) In some of these cases there is stoppage of the nose alternating with fluent coryza, and in still others there is a distressed stoppage at the bridges of the nose. There is one symptom of the nose that is usually considered to belong exclusively to Colchicum, viz.: patient cannot bear the smell or sight of food, but which Arsenic has. In that difficult disease to cure, difficult only because close individualization is neglected—chronic catarrh—this remedy will cure cases which have scabs in the nostrils, which when torn away leave the nostrils raw and bloody until other scabs are formed, also where there is a feeling in the nose as if he smelt sulphur alternating with that of pitch.

In neuralgia of the face many cases are curable by this drug only. In addition to the same kind of pains as elsewhere, violent burning relieved by heat, and with the characteristic mental symptoms we have a form with burning, stinging pains, as from red-hot needles. (Agar-mus. has as from ice cold needles.)

In sore mouths of both adults and infants we will often have occasion to use our remedy. The aphthæ become livid or bluish, and are attended by the prostration so characteristic of this drug.

In diphtheria we find a disease that occasionally requires Arsen. The pseudo-membrane is dry looking and wrinkled, the breath putrid, but the prostration, anguish, and restlessness will be the decisive indication.

The thirst of Arsen. is one that will guide you in many diseases, febrile, gastric, and others. The thirst is very great but gratifying, it increases disturbance in the stomach and bowels so that it is either vomited immediately (Phos. after it has become warm) or is only drunk in small quantities, but very often. There may be nausea or vomiting of blood, etc., but it is always attended by this thirst. In the stomach and bowels, as elsewhere, the pains are burning, relieved by heat. In cancer of the stomach, round ulcer, gastritis, and the like, Arsen. will often be indicated by the symptoms I have given, and if the disorganization is not too far advanced it will cure. In choleraic conditions, Asiatic, morbus, and infantum, always give Arsen. if the characteristic thirst, vomiting, and restlessness are present. In diarrhœa and dysenteries also the discharges are bad smelling, corroding whenever they touch, and prostrate the patient. Sometimes stools and urine are involuntary. (Hyos. and Crotalus horrid. also.)

For hemorrhoids we must sometimes use Arsenic to cure. These are such as have the stitching pains when walking or sitting, but not when at stool with the characteristic pain also.

In diseases of the urinary organs, atony of the bladder, no desire to urinate and no power to do so, seems to have lost all control over the power to discharge it. This condition we find more particularly after parturition. I once cured a case of enlargement of the prostate of six months' standing with Arsenic. In Bright's disease do not forget this remedy, for if indicated it will do good work.

In phagedenic chancres we may limit our choice to Arsen. and Merc. corr. Those requiring this remedy are of livid hue, with intense burning, even sloughing. A sufficient guide to the choice is found in the mental symptoms. With Merc. corr., however much patient suffers he is perfectly quiet and composed, which is in striking contrast to the anguish and restlessness of Arsen. Also chancres with gangrenous degeneration require Arsenic.

In diseases of the female genital organs from leucorrhœa up to cancer we should remember the characteristic symptoms of Arsen. In diseases of the ovaries, including tumors, remember the burning, restlessness relieved from heat, etc., and also that wherein she cannot keep her foot still with pain in corresponding leg, with Zinc she cannot keep her feet still—both of them.

Uterine hemorrhages in feeble women, cachectic, with disorganization of the uterus or ovaries, in fevers and when aphthæ breaks out, in labor when the vagina and soft parts seem very rigid and small. She has burning, throbbing, lancinating pains in the uterine region.

In diseases of children Arsenic has but few rivals. I have already spoken of cholera infantum, but in the complaints of teething we could not get along without this remedy. The child has undigested, fetid stools, and is emaciated. Its skin is dry and shriveled; it is restless, particularly after midnight; it has paroxysms of anguish day and night, during which it often strikes its forehead with its hand as though that afforded relief. (With Rhus it strikes its head against the wall or floor; with Helleborus it gets on its hands and knees and plunges forward, striking its head violently on the floor.) The child has the same thirst and vomiting as its seniors. The gums over the advancing teeth sometimes appear to be blistered (Apis, Silic.) or to be filled with a dark, watery fluid (Kreosote). Sometimes the child has a pale, waxy look (Apis) and is very weak. It wants to be carried very quickly. (When the same condition exists in croup Bromine is the remedy.) It cries much during and after nursing, and as soon as it eats anything; its diarrhœa is also aggravated from the same causes. It may vomit and have a stool at the same time. It is much exhausted every time it moves. There is often cold perspiration, reminding us of Verat. alb. With Ars. it is warm under the clothing, or when it first comes out, with Verat. it comes out cold, and both also have cold breath. In the various convulsions of children this is the remedy if the child lies as if dead, pale but warm, is breathless for some time, finally it twists its mouth first to one side and then to the other. A violent jerk appears to pass through the whole body and its respiration and consciousness gradually return. The child coughs if spoken to (Nat. mur. cries).

Consumption is a disease many cases of which are curable by Arsenic. Such are characterized by acute, sharp, fixed, or darting pains in the apex and upper third of the right lung. The night-sweats come on when patient first falls asleep and soon cease (Kali carb. also). With Phosh. the sweats begin as soon as he falls asleep and continue as long as he sleeps. The cough is followed by an increased difficulty in breathing; it is worse from one to two o'clock A. M. When coughing the pain extends into the thigh. The cough is excited by a sensation as from the fumes of sulphur, as from a lucifer match (Lycopodium), and by drinking (Kali carb.). The expectoration is frothy saliva, mucus streaked with blood, frothy substance like the beaten white of an egg, smelling like garlic. The hemorrhages of incipient phthisis which require this remedy are such as follow great loss of blood; burning heat all over, especially with pain between the shoulder-blades in drunkards or when the menses are suppressed. (Pulsatilla and Phos.) Asthma frequently requires Arsenic when the attacks come in the morning (evening, Pulsat.), when worse in windy weather (in thundering weather Silicea). Patient has to sit up in bed with knees drawn up and rests his hands and arms on his knees; it comes on from twelve midnight till two; he loses breath on every movement with anxiety and prostration; dyspnœa if he lies on his back; he has roaring in the ears and headache. In gangrene of the lungs with green, ichorous sputa.

In the heart where there is palpitation with anguish; cannot lie on back; worse on going up stairs. He has palpitation and tremulous weakness, after stool must lie down.

In angina pectoris give Arsen. if there is its characteristic anguish; the least motion makes him lose his breath; he sits bent forward or with head thrown back; worse at night and particularly after midnight. In dropsy of the heart with anguish, restlessness, etc.

In the limbs we find burning ulcers on the tips of fingers and toes, filled with blood on the ends of fingers, and ulcers and scabs under the nails. There is a peculiar paralysis which Arsen. cures, that of the right upper and left lower extremity. Terebinthina has a like condition, and Can. ind. has paralysis of the right arm and both legs. The patient has an uneasiness in the legs; cannot lie still at night; has to change position constantly or walk about to get relief. In these cases you must remember Rhus; the mental symptoms will differentiate between them. In the indolent ulcers of the leg Arsen. stands along with the other antipsorics—Calc. c., Lyc., Silic., and

Sulph. The pains are burning and lancinating, relieved by heat. I once cured an ulcer of twenty-five years' standing of a man in his sixties in eight weeks with two prescriptions of Arsen.$^{cc}$. Another form cured by this remedy is when spreading blisters start on soles of feet and toes and become ulcers.

Arsen. has a profound action on the nervous system, as is shown by the great prostration, restlessness, and anguish which accompany nearly all cases.

Many cases of sleeplessness which are caused by anguish, restless tossing about, will disappear immediately if this remedy is given.

In fevers of almost every kind, eruptive, malarial, typhoid, pyæmic, and inflammatory, Arsen. is a potent remedy. The anguish, restlessness, and prostration, relief from heat, the characteristic thirst and vomiting, are always present. Other symptoms are coldness and chill, renewed after eating and drinking. The sweat comes on some time after the heat or not at all. Burning heat with unquenchable thirst. From these symptoms we see that this is an important intermittent fever remedy. The indications are clear cut, and to give another remedy when Arsen. is indicated reveals an ignorance that is little less than criminal. He who gives the indicated homœopathic remedy in every case of intermittent fever will never need to injure his patient with Quinine.

Of the different classes of disease:

In septic changes in the blood, exanthemata, ecchymoses, petechiæ, decubitus, tissue changes with burning pains, chronic inflammation of serous membrane, with copious serous effusions (Apis). One point of difference you must always remember is that this drug is relieved by cold and Arsen. by heat. In inflammatory swelling with burning, lancinating pains, we must often give Arsen. Face, abdomen, and all the limbs, especially the legs, are dropsical. In these cases you must notice the difference between. Arsen. and Apis, Apocynum can., Digitalis, and others. We often find rapid emaciation with atrophy of the feet and finger tips. The ulceration of Arsen. constantly extends in breadth; with Silic. it is the reverse. The discharges from the mucous membranes, ulcers, and the like are acrid, burning, corroding, and often extremely offensive. In gangrene we must always remember Arsen.; here we must differentiate between this remedy and Carbo veg., Lach., and Secale cor. The difference between Arsen. and Carbo veg. is principally in the mental symptoms. Torpor of mind and body characterizes the latter, as excessive sensitiveness the former.

Lach. is distinguished by blueness of the affected parts and the aggravation from sleep, while Secale shows aggravation by heat and covering up warm, the direct reverse of Arsenic.

Arsenic is a specific for dissecting wounds. Also in poisoning from decayed or morbid animal matter, by inoculation, inhalation, or swallowing.

In diseases of the skin Arsen. plays a prominent part. It is required in the bran-like, dry, scaly eruptions, with itching and burning, the latter increased by scratching and followed by bleeding. Or there may be the parchment-like dryness of the skin, and black vesicles causing burning pain. In hemorrhagic measles and small-pox it is the remedy.

The complements of Arsen. are Allium sat., Carbo veg., and Phosph.

